# Supplementary material for: Efficiency and irreversibility of movements in a city
Source: Sci Rep. 2020 Mar 9;10:4334. doi: 10.1038/s41598-020-60744-6 (PMC7062822; doi:10.1038/s41598-020-60744-6)
Supplement: Supplementary file 1 — Supplementary Information. [file 41598_2020_60744_MOESM1_ESM.pdf]

Efficiency and irreversibility of movements in a city: Supplementary Information

Indaco Biazzo

Politecnico di Torino, Corso Duca degli Abruzzi 24, Torino, Italy

Abolfazl Ramezanpour*

Physics Department, College of Sciences,
Shiraz University, Shiraz 71454, Iran and
Leiden Academic Centre for Drug Research,
Faculty of Mathematics and Natural Sciences, Leiden University,
PO Box 9500-2300 RA Leiden, The Netherlands

(Dated: February 11, 2020)

* aramezanpour@gmail.com

I. SUPPLEMENTAL FIGURES

Here we report the results which mentioned in the main text but the reader is referred to the Supplemental Material for the figures.

We take a two-dimensional grid of $N = L \times L$ sites for $G(V, E)$ with connectivity $z = 4$ and links of length one. The population distribution (m_a) is constructed by simulation of the growth model described in the main text (Sec. II) [1] with parameters $m_{seed} = 1, c_0 = 1, r_0 = 1$. For a real city, the network structure G and population distribution are provided by the available data from [2, 3] (see Fig. 1 for an example). The OD mobilities are obtained from [4]:

$$m_{a\to b} = m_a p_{a\to b} = m_a \frac{m_b/M(r_{ab})}{\sum_{c\neq a} m_c/M(r_{ac})}. \tag{1}$$

Given the expected travel times $\tilde{t}_{ab}$, the flows F_{ab} are determined by the shortest path (in time) strategy. We shall assume that the starting time of the OD trips in the time interval ΔT_o obeys a centred Gaussian distribution of standard deviation $\Delta T_o/3$. The actual travel times are computed by

$$t_{ab}(F_{ab}) = t_{ab}(0)\left(1 + g(\frac{F_{ab}}{F_{ab}(0)})^{\mu}\right), \tag{2}$$

with $F_{ab}(0) = F_{ba}(0) = M/(2|E|)$. We also assume that $t_{ab}(0) = t_{ba}(0) = 1$ for all directed edges in G. Therefore, there is no structural asymmetry in the model. We consider a learning process in which the expected travel times are updated by using the information about the actual travel times in the previous cycle. More precisely, for cycle n we take $\tilde{t}_{ab}(n) = \lambda t_{ab}(n-1) + (1-\lambda)\tilde{t}_{ab}(n-1)$, with $\lambda = 1/2$ as a damping parameter and $\tilde{t}_{ab}(0) = t_{ab}(0)$. We repeat the cycle for $n_c = 20$ times and report the results at the end of this process.

A. Simulated population distributions

We start with the results which are obtained by the simulated population distributions. Figures 2 and 3 display the average efficiency and the average relative entropies when only one parameter g or ΔT_o changes. The KL divergence is divided by $\ln(2|E|)$ to be able to compare it for different city sizes. We see how much the two parameters contribute to

the behaviour of the system efficiency. For comparison, the figures also show the results obtained with no learning, that is without any knowledge of the actual travel times in the previous cycles. Note that learning dose not necessarily increases the efficiency because the aim of learning here is just to find the shortest (time) path. Moreover, as the figures show, the learning process considerably changes the behaviour of the efficiency with the KL divergence. The relation with the relative entropy ΔS_T, by contrast, does not qualitatively change by the learning process. The latter solely measures the changes in the size of the time intervals ΔT_d and ΔT_r which usually grow by increasing g or ΔT_o. On the other hand, the KL divergence is affected by both the size of the time intervals and the distribution of the arrival times in these intervals.

The behaviour of the efficiency with the relative entropies $D_{KL}(\overrightarrow{\mathbf{f}}\,||\,\overleftarrow{\mathbf{f}})$ and ΔS_T is shown in Fig. 4 for some independent realizations of the population distribution. We observe a considerable negative correlation between the efficiency and the relative entropies, except for the case $g = 0$, where the flows have no effect on the travel times. In this case, it is only the population distribution that determines the efficiency and the relative entropies. The positive correlation in this case is probably related to the fact that population distributions with closer ODs could result to smaller travel times T but larger divergences D_{KL} while the number of trips only changes slightly due to the small OD distances. Note that both the size of destination time interval ΔT_d and the distribution of arrival times in this interval affect the KL divergence whereas only the former is important for the relative entropy ΔS_T.

Dependence of the main quantities on ΔT_d is reported in Fig. 5 for the case $(g = 1, \mu = 2)$. The destination time interval ΔT_d plays a central role in this study; the network structure and the impact of the forward flows on the travel times usually give rise to a large ΔT_d (larger than ΔT_o). And the size of ΔT_d directly affects the divergence of the backward flows from the forward ones. Here both the forward and backward travel times T_{OD}, T_{DO} are expected to increase with ΔT_d. Therefore, as the figure shows, the total travel time and D_{KL} are positively correlated with ΔT_d. On the other hand, we observe in Fig. 5 that the total number of services C is not very sensitive to ΔT_d. This is true for both the forward and backward contributions C_{OD}, C_{DO} as long as ΔT_o and Δt are fixed (the latter here is set to one).

B. Real population distributions

Now, we report the results which are obtained by using the population distributions of 20 real cities. Figure 1 displays such an example for the core and commuting parts of a real city. The cumulative distribution of the simulated OD times T_{OD}, the normalized flows f_{ab}, and the destination time intervals (across the sites) of three cities are displayed in Fig. 6 for the core parts of the cities. Here, we observe a tendency to exhibit scale free behaviours by introducing the impact of the flows on the travel times.

In Fig. 7 we see how the average efficiency and the average relative entropies of these cities behave for various μ and g. Again, we observe that learning changes the sign of (η, D_{KL}) correlations whereas ΔS_T always shows a negative correlation with the efficiency. Figure 8 displays the averages η, D_{KL} and ΔS_T when the parameters g and μ are changing for a fixed ΔT_o. The data sets can well be described by an exponential relation $\eta \propto \exp(-\beta D_{KL})$, with exponent $\beta = 19.3 \pm 1.8$. Similar behaviour is also observed with the simulated population distributions in Fig. 2.

---

[1] Ruiqi Li, Lei Dong, Jiang Zhang, Xinran Wang, Wen-Xu Wang, Zengru Di, and H.Eugene Stanley , "Simple spatial scaling rules behind complex cities." NATURE COMMUNICATIONS 8: 1841 (2017).

[2] Eurostat population grid; *Available at* `http://ec.europa.eu/eurostat/web/gisco/geodata/reference-data/population-distribution-demography/geostat`.

[3] Center for International Earth Science Information Network CIESIN Columbia University C. Gridded Population of the World, Version 4 (GPWv4): Population Count. Palisades, NY: NASA Socioeconomic Data and Applications Center (SEDAC); 2016. Available from: `http://dx.doi.org/10.7927/H4X63JVC`.

[4] Yan X-Y, Zhao C, Fan Y, Di Z, Wang W-X. 2014 "Universal predictability of mobility patterns in cities." J. R. Soc. Interface 11: 20140834.

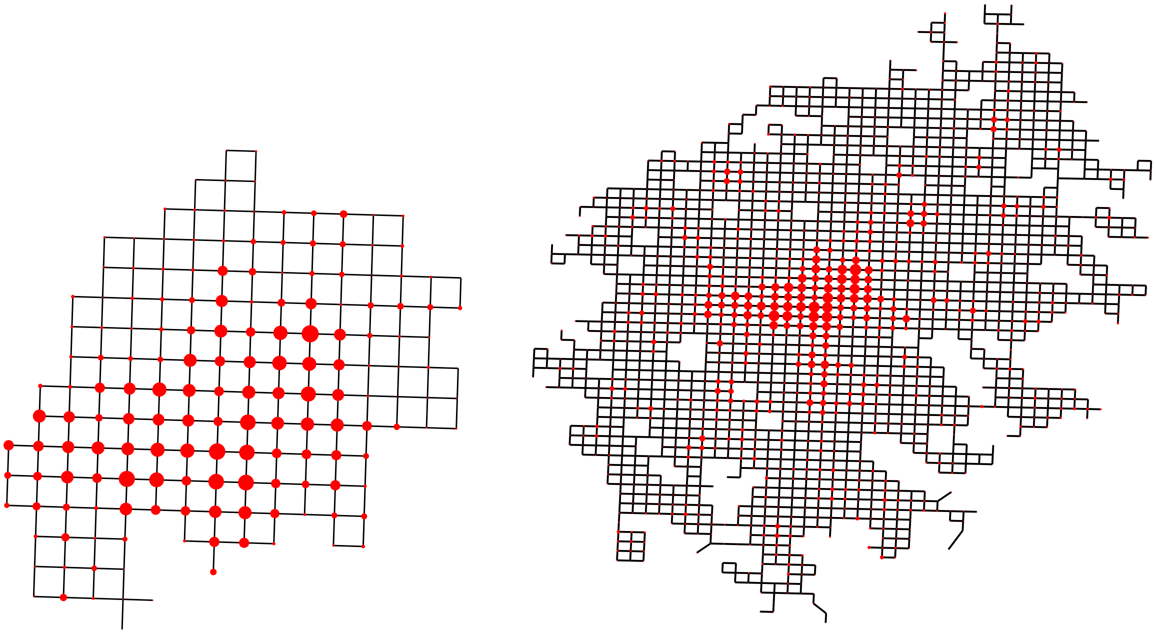

FIG. 1. An example of the population distribution in a city (Turin). Left: the core part of the city ($N = 169, M = 935534$). Right: the core plus the commuting part ($N = 1656, M = 1772227$). The raw data are from [2, 3] and here are plotted with networkx(2.2).

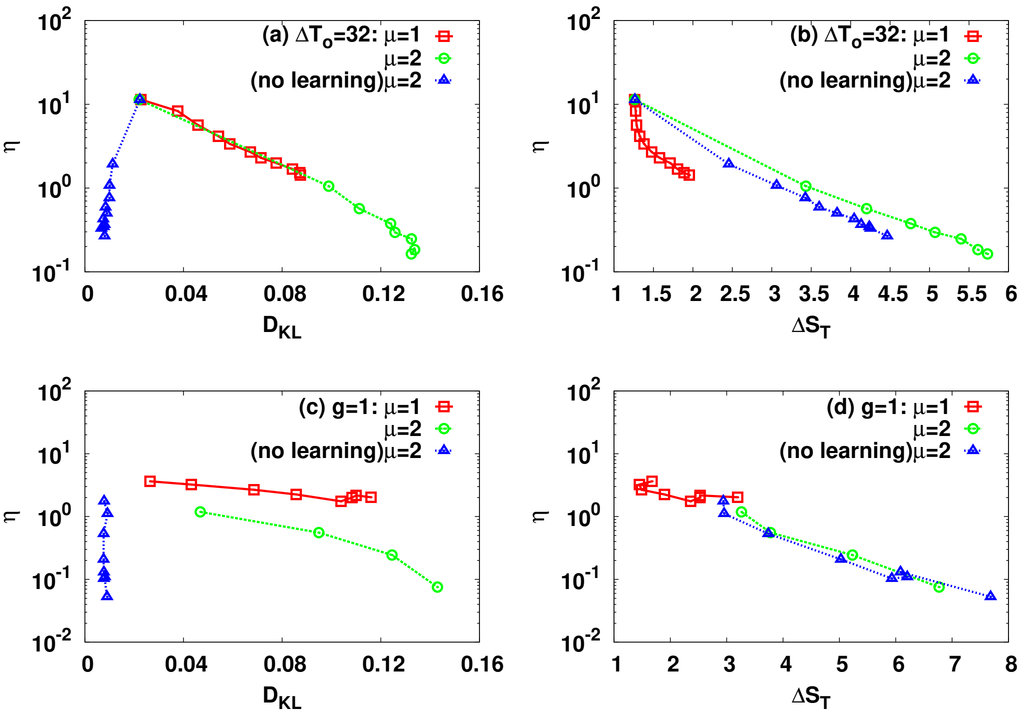

FIG. 2. The average efficiency (η) vs the average relative entropies ($D_{KL}, \Delta S_T$). Top: the behaviour when only $g \in (0, 2)$ changes with μ and ΔT_o fixed. Bottom: the behaviour when only $\Delta T_o \in (2^0, 2^6)$ changes with μ and g fixed. The average is taken over 100 realizations of population distribution and movements (with learning) on a two-dimensional grid of size $N = 50 \times 50$. The errorbars are about the point sizes.

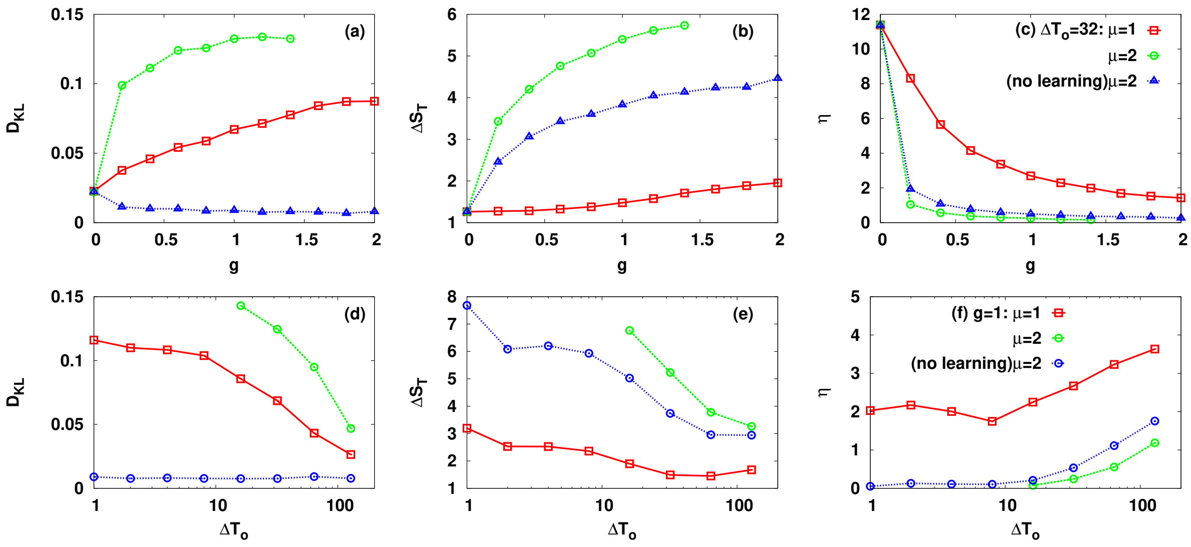

FIG. 3. Variation of the average efficiency and relative entropies with g and ΔT_o. Top: the behaviour when only g changes with μ and ΔT_o fixed. Bottom: the behaviour when only ΔT_o changes with μ and g fixed. The average is taken over 100 realizations of population distribution and movements (with learning) on a two-dimensional grid of size $N = 50 \times 50$. The errorbars are about the point sizes.

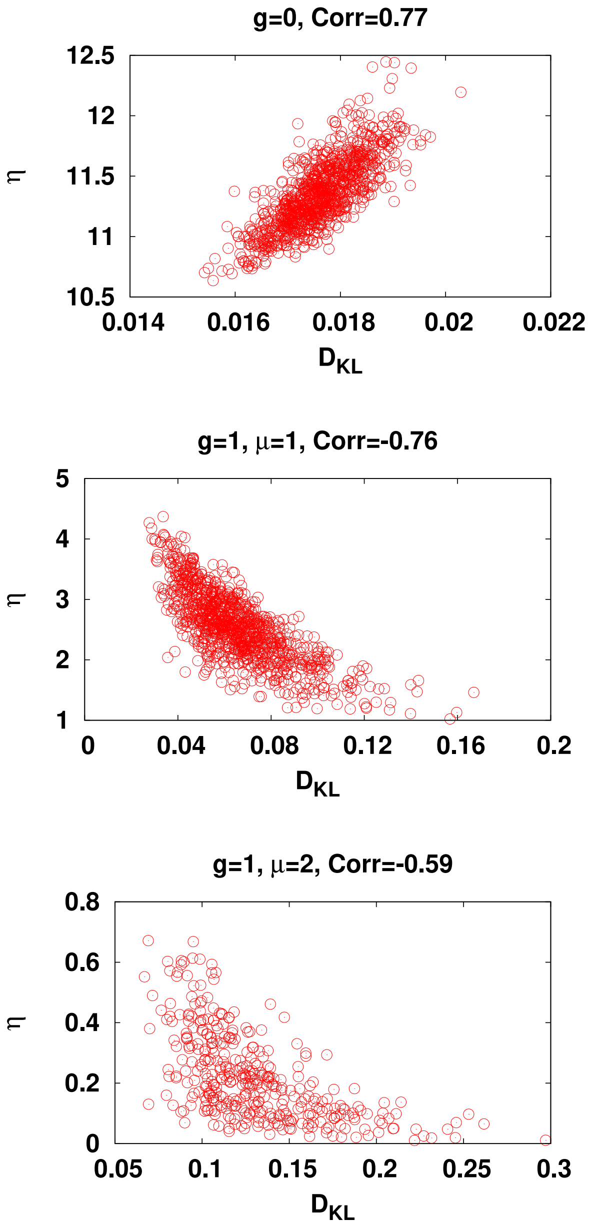

FIG. 4. The efficiency η vs the relative entropies D_{KL} and ΔS_T. Each point shows a realization of the simulated population distribution and movements (with learning) on a two-dimensional grid of size $N = 50 \times 50$. Here $\Delta t = 1$ and $\Delta T_o = 32$. The Pearson correlation coefficient shows the sign and magnitude of correlation between the two quantities.

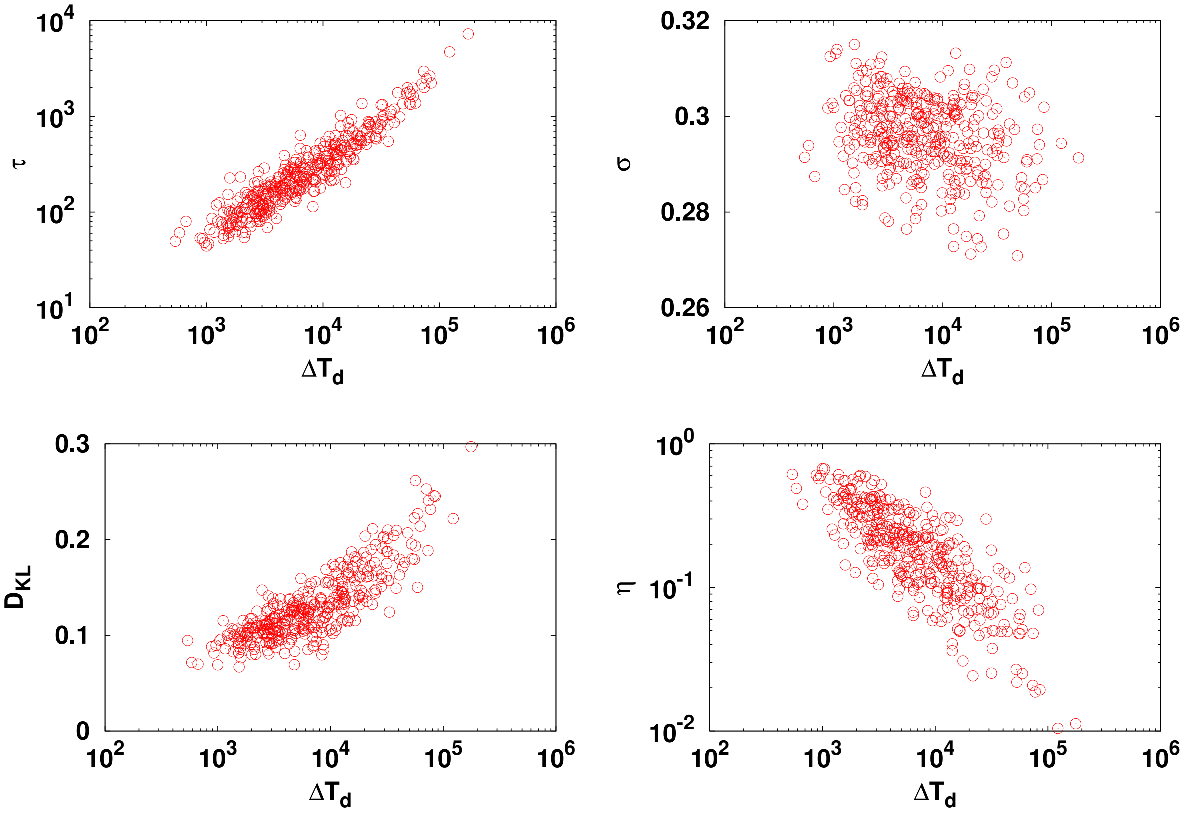

FIG. 5. Dependence on the destination time interval ΔT_d. The model parameters here are $\mu = 2, g = 1$ and $\Delta T_o = 32$. Each point shows a realization of the simulated population distribution and movements (with learning) on a two-dimensional grid of size $N = 50 \times 50$. As before $\Delta t = 1$.

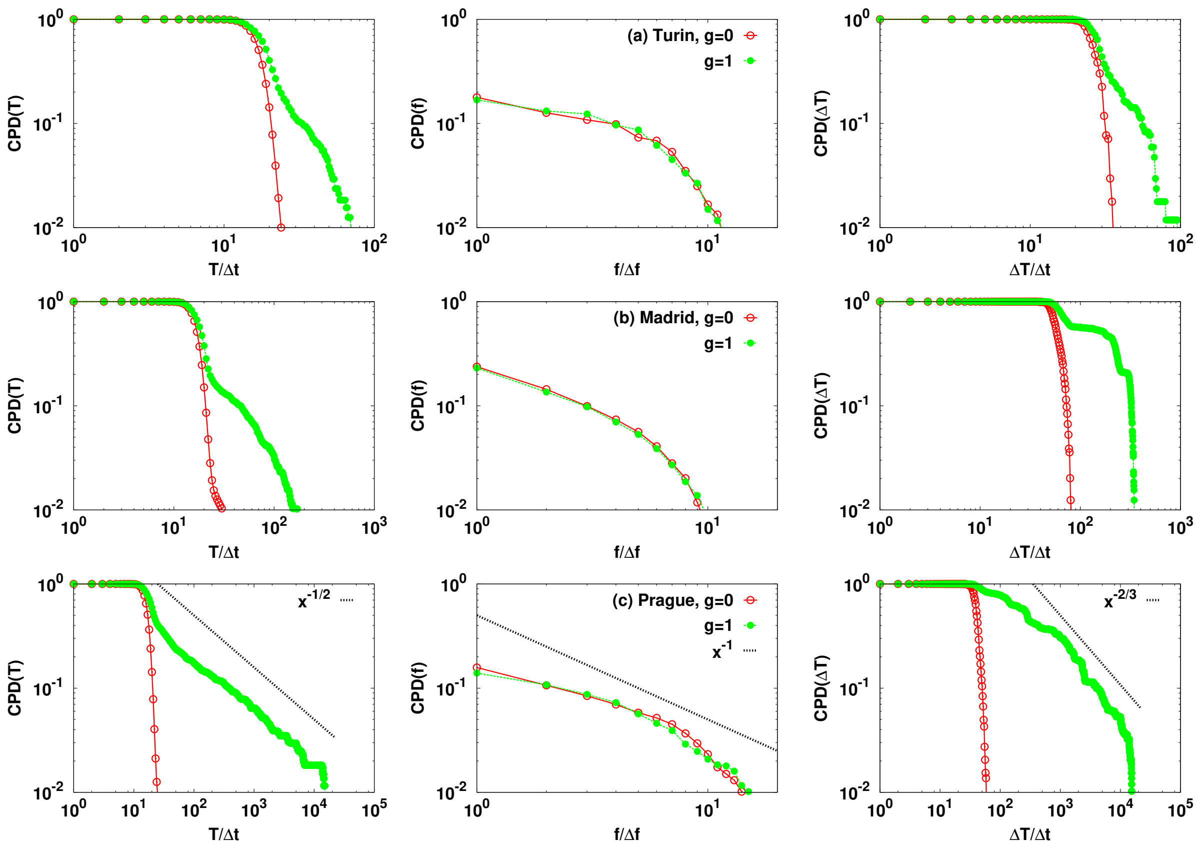

FIG. 6. Cumulative probability distributions of simulated T_{OD} (left), f_{ab} (middle), and ΔT_d (right) in three cities. The data show distribution of T_{OD} across the individuals i, f_{ab} across the directed edges (a, b), and ΔT_d across the network sites a. The data are obtained by numerical simulation of the movements after 20 learning cycles using the population distributions of the cities. The model parameters are $\mu = 3, g = 1, \Delta T_o = 32$ and $\Delta t = 1$. For comparison we also report the results for the case $g = 0$.

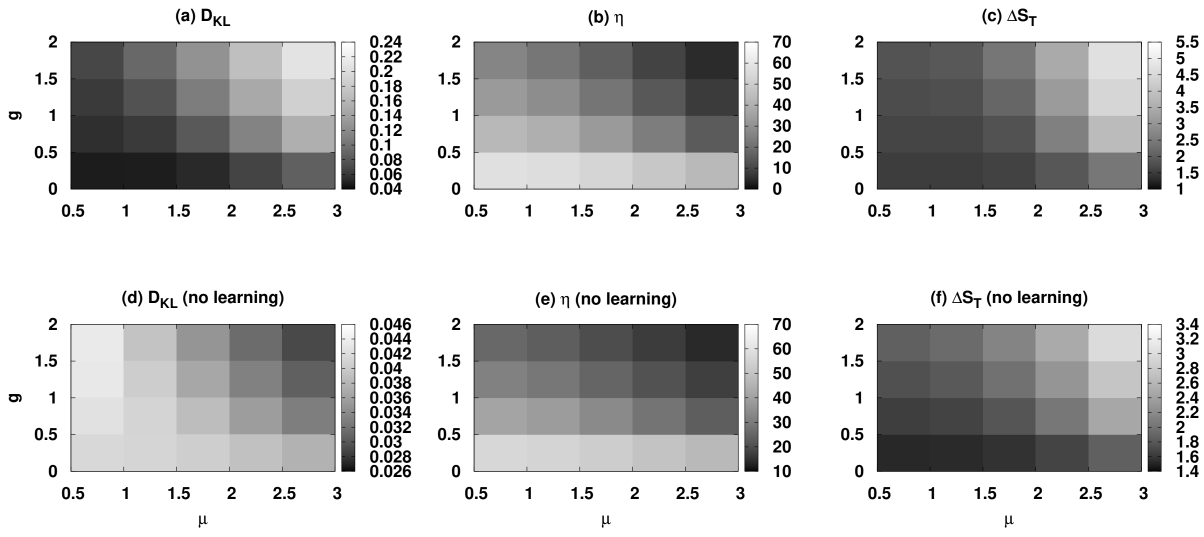

FIG. 7. The average behaviour of the efficiency (η) and the relative entropies ($D_{KL}, \Delta S_T$) in 20 cities (the core parts). The data are obtained by numerical simulation of the movements after 20 learning cycles using the population distributions of the cities. The average is taken over the cities for $\Delta T_o = 32$. For comparison we also report the results obtained without learning of the travel times (bottom panels).

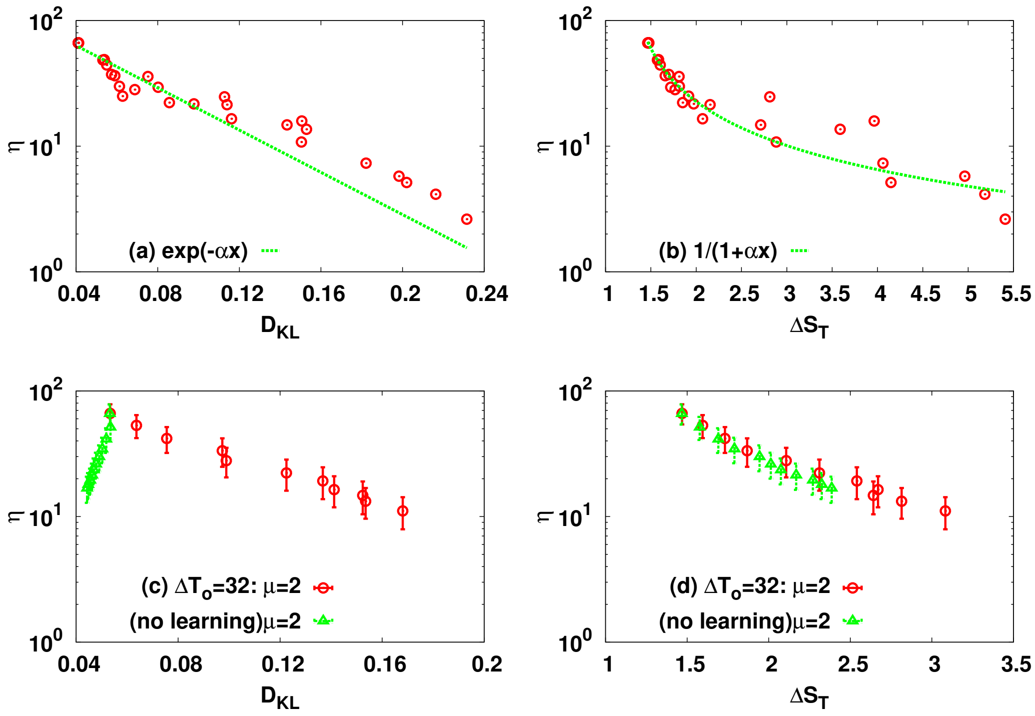

FIG. 8. The average efficiency (η) vs the relative entropies $(D_{KL}, \Delta S_T)$ for 20 cities (the core parts). Top: the behaviour for different values of $g \in (0,2)$ and $\mu \in (0,3)$ for a given $\Delta T_o = 32$ fixed. Bottom: the behaviour when only g changes with μ and ΔT_o fixed.
